# Influence of Silicate Modulus and Eggshell Powder on the Expansion, Mechanical Properties, and Thermal Conductivity of Lightweight Geopolymer Foam Concrete

**DOI:** 10.3390/ma18092088

**Published:** 2025-05-02

**Authors:** Mohamed Abdellatief, Mohamed Mortagi, Hassan Hamouda, Krzysztof Skrzypkowski, Krzysztof Zagórski, Anna Zagórska

**Affiliations:** 1 Department of Civil Engineering, Higher Future Institute of Engineering and Technology, Mansoura 35516, Egypt; 2 Faculty of Engineering, Mansoura National University, Mansoura 35516, Egypt; 3 Faculty of Engineering, Mansoura University, Mansoura 35516, Egypt; 4 Civil and Architectural Construction Department, Faculty of Technology and Education, Suez University, Suez 41522, Egypt; 5 Faculty of Civil Engineering and Resource Management, AGH University of Krakow, Mickiewicza 30 Av., 30-059 Kraków, Poland; 6 Faculty of Mechanical Engineering and Robotics, AGH University of Krakow, Mickiewicza 30 Av., 30-059 Kraków, Poland; 7 Research Centre in Kraków, Institute of Geological Sciences, Polish Academy of Science, Senacka 1, 31-002 Kraków, Poland

**Keywords:** SiO_2_/Na_2_O ratio, geopolymer foam, Al powder, eggshell powder, ternary precursor

## Abstract

To address the demands of the low-carbon era, this study proposed a solution by using eggshell powder (ESP), fly ash, and ground granulated blast furnace slag together with alkaline solution in the preparation of lightweight geopolymer foam concrete (LWGFC). The aim of this study is to investigate the influence of replacing precursor materials with 5–20% ESP on the expansion behavior, physical, mechanical characteristics, and thermal conductivity of LWGFC. Additionally, the study examines the effect of varying the silicate modulus (SiO_2_/Na_2_O ratios of 1.0, 1.25, and 1.5) on the properties of LWGFC. Incorporating ESP from 5% to 20% with a constant SiO_2_/Na_2_O ratio reduced the initial setting time, while a high SiO_2_/Na_2_O ratio controlled the setting time and expansion volume. The high SiO_2_/Na_2_O ratio decreased the porosity and enhanced the compressive strength of the LWGFC but increased the thermal conductivity. The inclusion of more than 10% ESP content negatively affected compressive strength; however, a high SiO_2_/Na_2_O ratio can mitigate this detrimental effect. The thermal conductivity of optimal-content ESP mixtures with a SiO_2_/Na_2_O ratio of 1.0 was about 0.84 W/m·K, which is 2.1% lower than mixtures with a ratio of 1.25 and 18.6% lower than those with a ratio of 1.5. High-content ESP mixtures had a density of 1707 kg/m^3^, 0.97 W/m·K, and a compressive strength of 18.9 MPa at a low SiO_2_/Na_2_O ratio. Finally, the inclusion of ESP in the LWGFC, along with the use of an appropriate silicate modulus, resulted in improved strength development while decreasing porosity.

## 1. Introduction

Thermal insulation materials that are cost-effective and have a minimal environmental impact have gained worldwide attention as a solution for lowering energy use and carbon emissions during the entire lifecycle of buildings [1,2]. It is commonly recognized that these materials include organic and inorganic materials [3,4]. Compared to organic materials such as expanded polystyrene, foam concrete (FC), which often consists of cement, tap water, and foam agents, is a promising kind of inorganic lightweight material [5,6]. It is possible to produce a porous FC with a controlled density ranging from 300 kg/m^3^ to 1800 kg/m^3^, making it a promising thermal insulation material for energy-efficient construction. Unfortunately, the main precursor of FC is cement, and its manufacturing emits about 28% of CO_2_ emissions, contributing to significant ecological issues [7,8]. Therefore, the geopolymer system is suggested as an appropriate alternative to cement, not only because it decreases CO_2_ emissions [9] but also due to its superior thermal insulation properties, which are critical for lightweight materials like foam concrete. Geopolymers have the potential to recycle a variety of by-product materials [e.g., fly ash (FA), slag (GGBS), and agricultural waste] as precursor materials, further enhancing their sustainability [10,11]. Geopolymer foam concrete (GFC), also known as foamed, aerated, cellular, or porous geopolymer, is created by introducing pores into the geopolymer slurry or mortar [12]. Due to the high strength of geopolymers, GFC offers lower density while maintaining comparable compressive strength and enhanced thermal insulation properties compared to traditional foam concrete (FC) [12]. Additionally, reducing the concentration of the alkali-activated solution and increasing the discontinuous gel structures within the geopolymer matrix further enhances its thermal insulation performance, making GFC a viable solution for sustainable thermal insulation applications [13]. Dang et al. [14] explored the influence of precursor materials and foam percentage on the density, strength, and thermal conductivity of GFC. They achieved a density range of 600 to 1600 kg/m^3^ for a 28-day compressive strength of 22.0 to 51.0 MPa, along with a thermal conductivity ranging from 0.15 to 0.48 W/m·K. Hao et al. [15] prepared GFC with a density range of 300 kg/m^3^ to 1200 kg/m^3^, which corresponded to strengths ranging from 0.5 MPa to 45.0 MPa. Raj et al. [16] studied the influence of the precursor content, water/binder ratio, SiO_2_/Na_2_O ratios, foam volume, and alkali percentage on the density and mechanical properties of GFC. They demonstrated that the SiO_2_/Na_2_O ratio, the precursor content, and the foam volume were the main factors influencing its density and strength performance. Additionally, conventional lightweight geopolymer foam concrete (LWGFC) faces challenges including slow strength development due to low calcium fly ash, dependency on corrosive alkali activators, foam instability causing uneven pores, durability issues in harsh environments, and limited use of alternative waste materials. These factors hinder its mechanical performance, cost-effectiveness, and sustainability.

Eggshells (ESs), the hardy outer layer of eggs, are one kind of agricultural waste, constituting about 10% of the mass of each egg, with over eight million tons of ESs expected to be produced annually due to the steady increase in global egg production [17,18]. In construction, ESs have been utilized as a partial cement replacement or filler in concrete to enhance sustainability and reduce environmental impact [19,20,21], while also finding applications as an adsorbent for ionic pollutants [22], a raw material for biodiesel [22], and a precursor for calcium phosphate ceramics [23]. One of the most significant chemical components of eggshells (ESs) is CaCO_3_, which provides a source of calcium that can participate in the formation of hybrid gels in geopolymer systems [24]. In geopolymers, the calcium from CaCO_3_ can partially substitute sodium in sodium-aluminum-silicate-hydrate (N-A-S-H) gels, forming calcium- (sodium) aluminum-silicate-hydrate C-(N)-A-S-H or calcium-aluminum-silicate-hydrate (C-A-S-H) gels, which enhance early strength development and improve the microstructure, as observed in fly ash (FA) and ground granulated blast-furnace slag (GGBS)-based geopolymers [24,25]. As a result, ESP can be used as a sustainable additive in geopolymer-based materials like LWGFC, reducing the environmental impact associated with ES waste disposal and excessive use of traditional precursors. For example, Zaid et al. [26] investigated the impact of eggshell powder (ESP) and nano-silica on the properties of high-strength concrete, finding that the ideal proportions of ESP and nano-silica were 5% and 10%, respectively. More recently, efforts have been made to incorporate ESP into foam concrete as a solution to the challenge of managing eggshell waste, while preserving the essential qualities needed for producing cement-based materials [27]. According to the previous studies [28,29], the main reaction product in FA-based GPC was N-A-S-H gel, regardless of heating treatment. On the other hand, GGBS was also employed as a precursor material owing to its Ca^+^ content, which contributed to its high early compressive strength. Recent studies [30,31] have indicated that the reaction products of GGBS-based geopolymer concrete can form Calcium silicate hydrates (CSH or C-S-H) and C-(N)-A-S-H gels. Additionally, Ca^+^-rich precursor materials were used to produce GPC with high strength, which improved the microstructure [31]. Several studies [32,33] have confirmed the creation of C-S-H, C-A-H, and C-(N)-A-S-H gels in the GGBS/FA-based GPC. Tekin [34] confirmed the formation of Ca-Si-O-Na and Ca-Si-Al-O-Na phases in a geopolymer paste made from Ca+-rich marble waste, which has a mineralogical composition similar to that of eggshell powder (ESP). Shekhawat et al. [21] investigated the microstructure of ESP/FA-based geopolymers, where they replaced 30% to 70% of the fly ash (FA) with ESP, varied the SiO_2_/Na_2_O ratio (0.5 to 2), and adjusted the curing temperature (25 to 80 °C) to produce the geopolymer paste. The increase in thermal curing temperature changed the microstructure of the ESP/FA paste. Additionally, the generation of Na_n_-(-Si-O-Al–O–Si–O–Si–O–)_n_– was demonstrated in the optimum geopolymer as a SiO_2_/Na_2_O ratio higher than one. Consequently, the incorporation of Ca^+^-rich ESP results in an enhancement of hydration products along with precursor gel. To the authors’ knowledge, the influence of ESP on the properties of lightweight geopolymer foam concrete (LWGFC) has not been systematically explored. Therefore, this study aims to investigate the effects of ESP content (ranging from 5% to 20%) and varying SiO_2_/Na_2_O ratios (1.0, 1.25, and 1.5) on the properties of LWGFC. The research first focuses on examining the fresh properties, followed by an evaluation of the hardened performance, which includes water absorption, porosity, thermal conductivity, and compressive strength. Finally, the morphological changes in the prepared LWGFC samples are analyzed.

## 2. Materials and Methods

### 2.1. Materials

**Figure 1:**
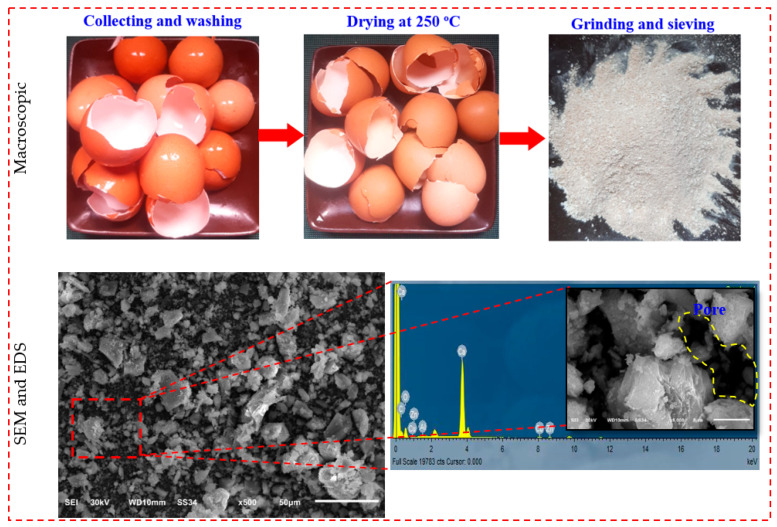
Macro and micromorphology of the ESP.

In the current investigation, ternary precursors GGBS, SF, and FA, in addition to employing eggshell powder (ESP), were utilized to produce the LWGFC. The specific gravity and a specific surface area for GGBS, SF, and FA were 2.89 and 396 m^2^/kg, 2.22 and 17,900 m^2^/kg, and 2.31 and 541 m^2^/kg, respectively. The chemical compositions of GGBS, SF, FA, and ESP were investigated using the XRF test, and the results are presented in Table 1. The preparation of ESP involved a multi-step process, as illustrated in Figure 1. Initially, eggshell waste was collected and thoroughly washed with tap water to remove surface impurities and organic residues (Figure 1). The cleaned eggshells were then heated at 250 ± 5 °C for 4 h to eliminate moisture and decompose organic components such as membrane residues and proteins (Figure 1, Heating at 250 °C) [24,35]. Finally, the dried eggshells were crushed, ground, and sieved to achieve a fine powder with a maximum particle size of 75 µm, as confirmed by SEM and EDS analysis showing the micromorphology and elemental composition of the ESP (Figure 1, Grinding and Sieving; SEM and EDS). It has been found that the main components of ESP are C, O, and Ca, and that ESP’s surface naturally contains pores. Based on the chemical composition reported in Table 1, it shows that ESP has a greater than 90% CaO. The physical properties of ESP in terms of specific surface area, specific gravity, and pore diameter are 3900 m^2^/kg, 0.85, and 22.80 µm, respectively. The average ESP particle size was determined to be 13.26 μm, slightly smaller than that of GGBS, following sieving through a 75 µm sieve (No. 200 mesh) using a vibratory sieve shaker to ensure particle size control and consistency. GGBS had a large amorphous hump with no distinguishable crystalline peaks, whereas ESP had a prominent peak at the diffraction angle of 2θ = 29.0°. This is the CaCO_3_ diffraction angle, which agrees with the XRF test’s results regarding chemical composition [24].

To produce the alkaline-activation solution (AAS), a commercialized sodium silicate (Na_2_SiO_3_) solution which contains 11.22% Na_2_O, 22.23% SiO_2_, and 66.65% H_2_O by weight, tap water, and NaOH, were used. The NaOH of an industrial level was 97 ± 1% pure in the flake form, which was dissolved in tap water to achieve a 12-M NaOH solution. The LWGFC samples can be designed by adjusting the AAS using both chemical components and different silicate moduli. As a foaming agent, aluminum (Al) powder, which has an average particle size of 55 μm, was utilized. Al powder was added to the LWGFC mixes at a 0.9% ratio by weight of the precursors (fly ash, GGBS, and ESP combined). As the fine aggregate, clean river sand with a specific gravity of 2.61 and a diameter size of less than or equal to 4.75 mm was used. Dolomite with an irregular shape was used as the coarse aggregate with a specific gravity of 2.56.

### 2.2. Mix Proportions and Sample Preparation

**Figure 2:**
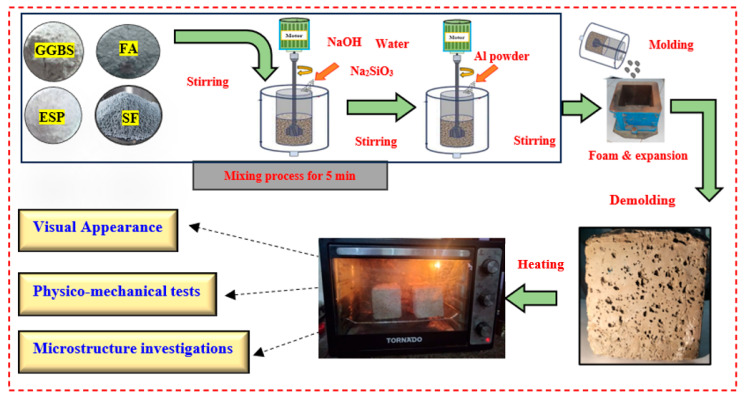
Mixing procedure of LWGFC incorporated with ESP.

This study focused on analyzing the impact of using 5–20% by weight of ESP to replace GGBS. Moreover, the effect of different SiO_2_/Na_2_O ratios (1.0, 1.25, and 1.5) on the microstructure and thermal conductivity of LWGFC were also examined. The mix proportions of the prepared LWGFC mixtures are shown in Table 2. Twelve LWGFC mixtures were designed and performed in this study. Figure 2 depicts the fabrication and preparation methods used for LWGFC. The mixtures were then poured into cubic molds with 100 mm × 100 mm × 100 mm dimensions. The freshly produced mixture rapidly grew within limited times, up to a few seconds, forming bubbles due to the reaction between Al powder and AAS. To minimize evaporation from the outer surface, the molds were covered with plastic. Following demolding, all samples underwent an additional one-day curing process in an oven at 60 °C. Subsequently, they were stored under controlled conditions at 25 ± 2 °C with a relative humidity of 60 ± 5% until the designated testing day.

### 2.3. Characterization and Analytical Methods

#### 2.3.1. Fresh and Mechanical Performance

The setting time of the LWGFC mixtures was assessed following ASTM C403/403M-16 [36]. Immediately after mixing, 100 × 100 × 100 mm^3^ cube samples were used to calculate both the fresh density and volume deformation. The fresh density of the LWGFC mixtures was measured in accordance with ASTM C567/C567M-19 [37]. The expansion of fresh LWGFC samples has a significant effect on their internal pore structure and durability. This expansion occurs due to the generated hydrogen, which depends on the Al powder content in the mixture as presented in Equation (1) [38].

2Al + 2NaOH + 2H_2_O → 2NaAlO_2_ + 3H_2_ (1)

Hajimohammadi et al. [29,39] reported that the Al inclusion modifies the geopolymerization kinetics by decreasing the time to peak. The Al reaction with the AAS rapidly releases aluminate ions that change the geopolymer gel network formation in the first few hours of reaction in comparison with non-foamed compositions. Therefore, the expansion height and relative expansion rate were used to evaluate the volume deformation of LWGFC. The physical properties of LWGFC, such as water absorption and dry density, were evaluated following ASTM C1585 [40] and ASTM C138 [41], respectively. Additionally, the compressive strength of the LWGFC was tested in accordance with ASTM C109 [42]. For each LWGFC mix design, a total of three replicate specimens were prepared and tested for each experimental property to ensure data reliability and reproducibility. The reported values in the manuscript represent the average (mean) values obtained from these replicates. Additionally, the standard deviation (SD) was calculated and included in the data analysis to assess the variability and consistency of the results.

#### 2.3.2. Microstructure Investigation and Thermal Conductivity

The morphology of the LWGFC samples was examined using scanning electron microscopy (SEM). Prior to analysis, the samples were dried and treated with isopropyl alcohol. To improve conductivity, a thin gold coating was applied using an ion sputtering coater. Mercury intrusion porosimetry (MIP) tests were conducted on 15 × 15 × 15 mm^3^ samples to determine their total porosity. Additionally, the thermal properties of the LWGFC were assessed using the Hot Disc TPS 2500 S thermal constants analyzer (Göteborg, Sweden), which has a resolution of 0.0001 W/m·K, in accordance with ASTM E1952-17 [43].

## 3. Results and Discussion

### 3.1. Expansion of LWGFC

**Figure 3:**
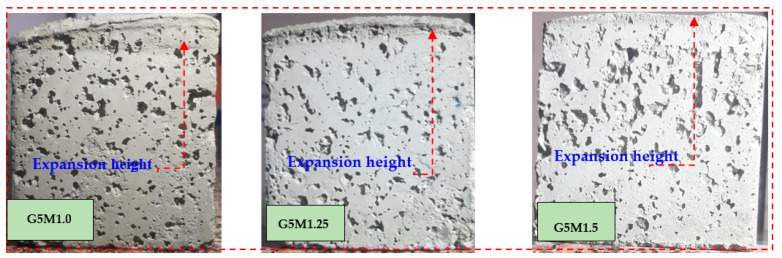
Expansion height of LWGFC containing ESP with different SiO_2_/Na_2_O ratios.

**Figure 4:**
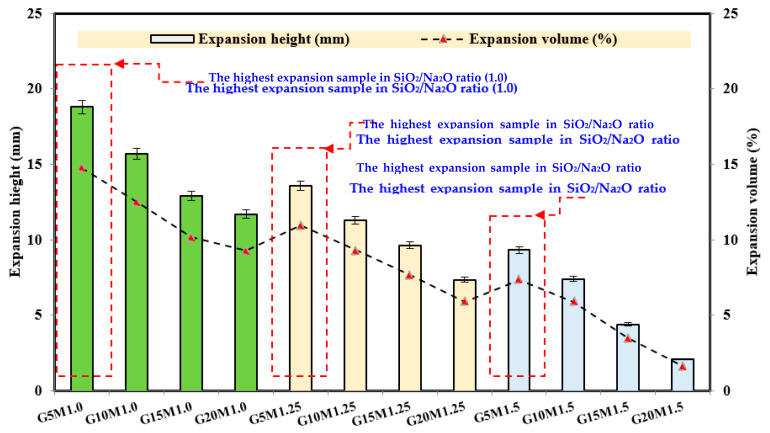
Effect of SiO_2_/Na_2_O ratio and ESP on the expansion height and volume of LWGFC.

The expansion volume and height of LWGFC were measured as shown in Figure 3 and Figure 4. The expansion volume and height of LWGFC were measured under varying ESP contents and different SiO_2_/Na_2_O ratios. High expansion volumes recorded at the low SiO_2_/Na_2_O ratio (1.0) were about 14.7%, 12.5%, 10.2, and 9.3% when incorporating ESP contents of 5%, 10%, 15%, and 20%, respectively. Equation (2) was used to estimate the expansion volume [38].

(2) $$Expansion\;volume\left(\%\right)=\frac{F_{v}-O_{v}}{F_{v}}\times 100$$

where *F_v_*: the final volume after hydrogen gas released (mm^3^) and *O_v_*: original volume before expansion (mm^3^).

As presented in Figure 4, the expansion height of LWGFC at a constant SiO_2_/Na_2_O ratio was reduced with increasing ESP. For example, the G5M1.0 sample had an expansion height of about 18 mm, while the G20M1.0 sample reduced the expansion height by 37.8%. According to Beghoura et al. [38], the fineness of the precursor affects the expansion volume of LWGFC, which should speed up the dissolution reactions and increase the speed at which hydrogen is released. Hamada et al. [44] confirmed that the high calcium ESP led to accelerating the geopolymerization reaction, which resulted in a shorter setting time and a decline in the expansion process. The expansion height is also influenced by the SiO_2_/Na_2_O ratio, which can be observed since the constant content of ESP leads to a decrease in the expansion height (Figure 3). Therefore, the low SiO_2_/Na_2_O ratio helps to expand the mixture, which may be related to the geopolymerization reaction as recommended by [45]. Furthermore, the G10M1.25 and G10M1.5 samples reduced the expansion height by 28.1% and 52.8%, respectively, compared to the G10M1.0 sample. Overall, the SiO_2_/Na_2_O ratio of the 1.5 group has high volume stability compared to other mixtures. Additionally, with increasing ESP content, the expansion volume of this group also decreased (Figure 4).

### 3.2. Setting Time and Density

**Figure 5:**
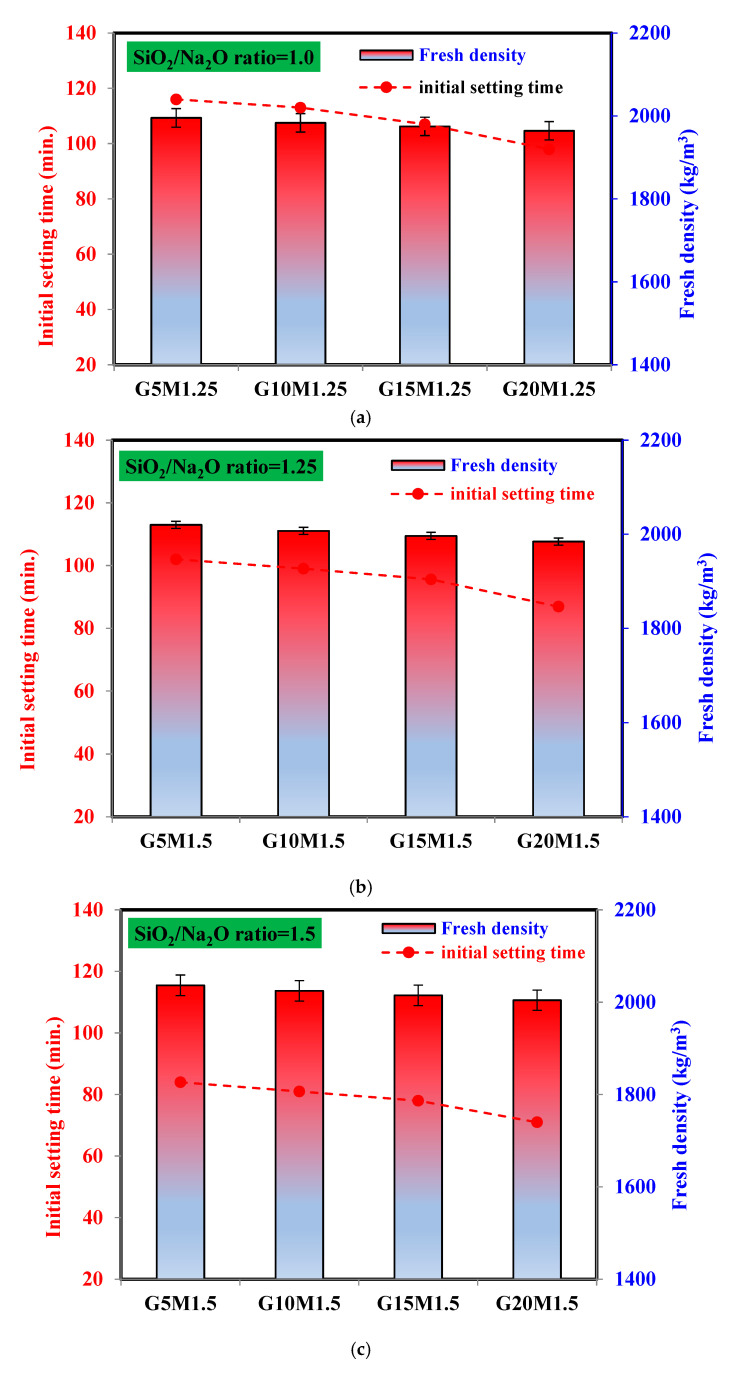
Setting time and density of LWGFC versus SiO_2_/Na_2_O ratio of (**a**) 1.0, (**b**) 1.25, and (**c**) 1.50.

Figure 5 presents the initial setting time and fresh density of LWGFC. Variations in ESP replacement levels and SiO_2_/Na_2_O ratios significantly influence both parameters. It is widely acknowledged that the production of porous materials relies on the balance between the gas formation rate and the setting characteristics of the matrix. As shown in Figure 5, the initial setting time decreases with the increasing SiO_2_/Na_2_O ratio. The reduction may be related to the hydration kinetics (dissolution, polycondensation, and geopolymerization reactions) of LWGFC [14,45]. It was also obvious that the setting time of LWGFC at the same SiO_2_/Na_2_O ratio also decreased with increasing ESP content, which may be related to the high content of calcium in eggshell particle resulting in an accelerated geopolymerization reaction. For instance, at the SiO_2_/Na_2_O ratio of 1.0, the initial setting time of LWGFC was shortened by 2.6%, 7.8%, and 15.5%, with incorporating 10%, 15%, and 20% ESP as a precursor compared to the 5% replacement, respectively. This suggests that the setting time was somewhat affected by the existence of ESP. This consideration can be demonstrated by the change in hydration kinetics caused by high-water absorption and the high rich-calcium content of ESP, in contrast to the high-activity of GGBS. Additionally, at the constant ESP content, it was also found that the setting time was reduced with the increasing of the silica concentration. For instance, at the 20% ESP content, adjusting the SiO_2_/Na_2_O ratio from 1.0 (Figure 5a) to 1.25 (Figure 5b), and 1.5 (Figure 5c) in the LWGFC samples drops the setting time to 86 min and 71 min, respectively. Using a high SiO_2_/Na_2_O ratio causes the LWGFC with a high calcium content to become more alkaline, which speeds up the geopolymerization reaction process and generates an early structure. Therefore, the current results confirm that the acceleration of initial setting time is directly proportional to the ESP content and SiO_2_/Na_2_O ratio. Contrarily, the fresh density of the LWGFC increased with the increment of the SiO_2_/Na_2_O ratio, as presented in Figure 5. The fresh density of LWGFC samples containing 20% ESP increased from 1964 kg/m^3^ to 1984 kg/m^3^ with a SiO_2_/Na_2_O ratio of 1.0 to 1.25. This trend can be associated with the increasing SiO_2_ concentration being able to react with the dissolved Ca, Al, and Si from the ESP and other precursors, which also accelerates the reaction products, leading to a denser microstructure, as we will discuss later [46]. Additionally, the addition of ESP influenced the fresh density of LWGFC (Figure 5), leading to a reduction ranging from 0.6% (1983 kg/m^3^) to 1.55% (1964 kg/m^3^) with a 10% to 20% replacement at a constant SiO_2_/Na_2_O ratio of 1.0, compared to a 5% replacement. This decrease can be attributed to the lower specific gravity of ESP particles compared to GGBS.

### 3.3. Morphology of LWGFC

**Figure 6:**
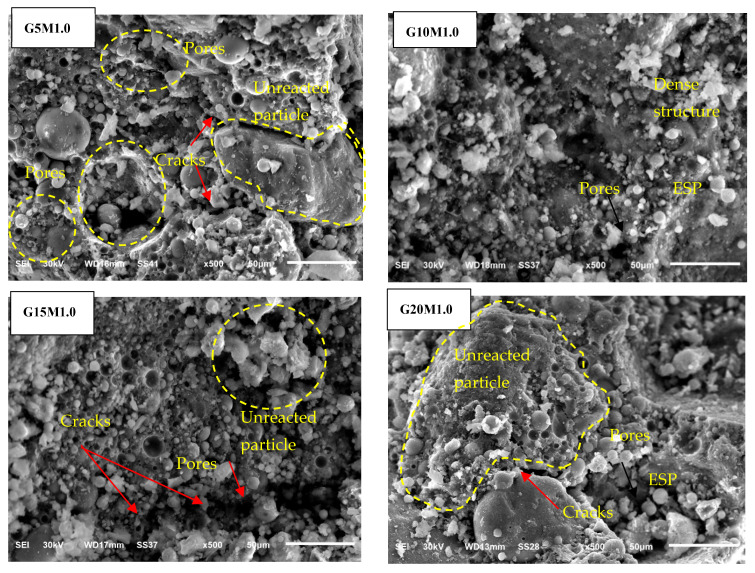
Morphology of LWGFC containing ESP at a SiO_2_/Na_2_O ratio = 1.0 at 50 µm.

**Figure 7:**
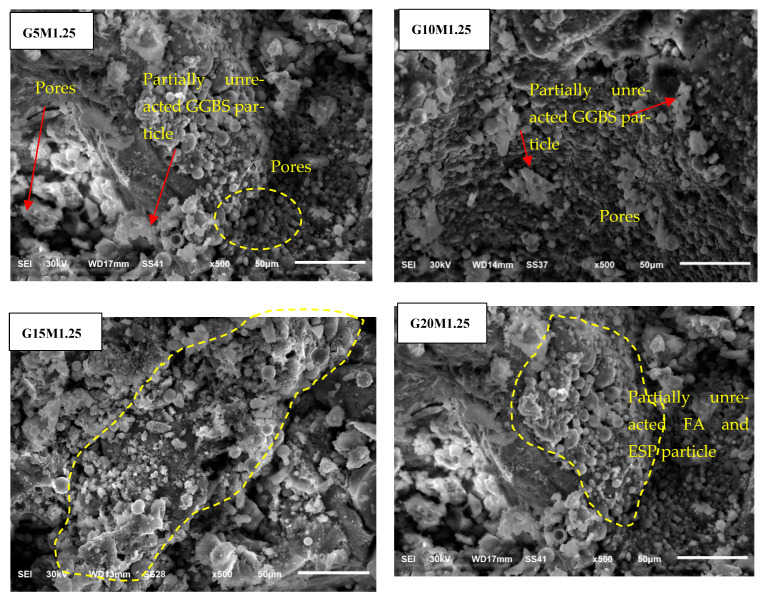
Morphology of LWGFC containing ESP samples at a SiO_2_/Na_2_O ratio = 1.25 at 50 µm.

**Figure 8:**
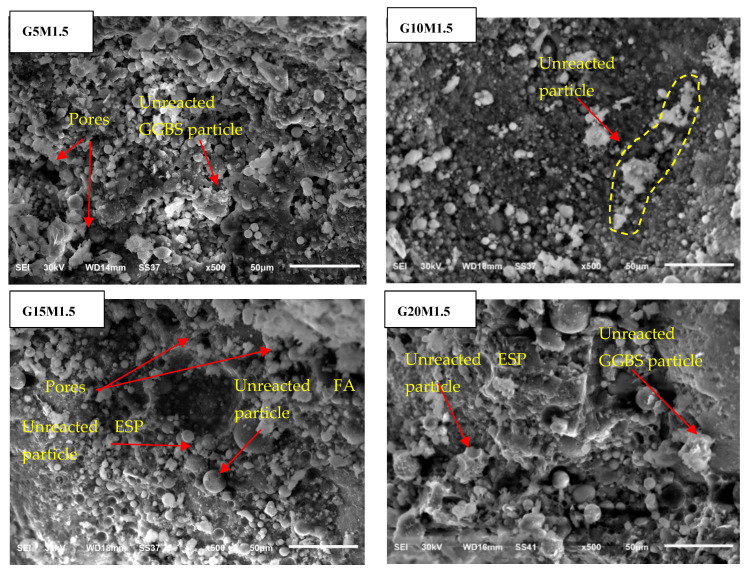
Morphology of LWGFC containing ESP at a SiO_2_/Na_2_O ratio = 1.5 at 50 µm.

Figure 6, Figure 7 and Figure 8 present the effect of ESP and the SiO_2_/Na_2_O ratios on the microstructure of the LWGFC. Generally, the morphology of the LWGFC at the same SiO_2_/Na_2_O ratio was altered with the addition of ESP. As illustrated in Figure 6 (at a SiO_2_/Na_2_O ratio of 1.0), the material exhibits a cellular structure with varying pore sizes. The microstructure appears to be weak due to low composite packing and the presence of larger pores, particularly at a 5% replacement level. Additionally, the G20M1.0 sample displayed a higher number of cracks at the interface between the geopolymer matrix and unreacted particles. These cracks likely formed during the foam preparation and curing stages, potentially resulting from differences in the thermal expansion coefficients of aluminum particles, the high-water absorption capacity of ESP, and geopolymerization reactions. In contrast, the G10M1.0 sample exhibited a relatively homogeneous morphology with smaller pores and a denser structure compared to the other samples. Further, a few cracks and pores can be found on the pore surface of the G15M1.0 mixture (Figure 6). The morphology of LWGFC reveals that ESP particles effectively interlock with the geopolymer gel and filler, refining the pore structure and enhancing the bonding between components to form a dense network. As the SiO_2_/Na_2_O ratio increases to 1.25 and 1.5, the presence of larger pores is noticeably reduced compared to lower SiO_2_/Na_2_O ratios, as depicted in Figure 7 and Figure 8. Consequently, this reduction in the pores decreases the porosity of the LWGFC, which tends to decline with the increase in ESP content. These results may be associated with the kinetic reactions of the high calcium content binder (GGBS+ESP) in the LWGFC at high alkaline activators, which redistribute the pore size in the matrix.

Similarly, the changes in precursor substitution and SiO_2_/Na_2_O ratios altered the morphology of LWGFC samples, especially at a low activator modulus. This degree of alteration is proportional to the SiO_2_ concentration. For the G10M1.0 sample, the unreacted ESP particles were easily seen. These particles interlocked with the SF and FA particles in a jagged shape and were lower than GGBS particles. The G10M1.25 (Figure 7) and G10M1.5 (Figure 8) samples also had a geopolymer matrix with small pores and a few unreacted GGBS and FA particles, which were lower than the G10M1.0 sample. Therefore, a homogenous geopolymerization matrix was found in the case of high SiO_2_/Na_2_O ratio (G10M1.5 sample), and the structure became denser and more compact. The explanation for this phenomenon is that the high dissolution of GGBS and ESP significantly refined the pores in LWGFCs by increasing the SiO_2_/Na_2_O ratio from 1.0 to 1.50, resulting in a higher heat release that enhanced the geopolymerization process. Additionally, the mixture with low alkalinity and high ESP replacement shows a noticeable presence of unreacted GGBS and ESP particles. These unreacted particles may contribute to the increased calcium (Ca) content observed in the mixture. Previous studies demonstrated that more C-A-S-H hydration gels transformed into N-A-S-H when GGBS was replaced by waste materials with a high Ca concentration and a majority of Si and Al components [28,31]. Especially for 10% replacement ESP, a low amount of unreacted ESP in LWGFC can be found in Figure 7 (G10M1.25 sample) and Figure 8 (G10M1.5 sample) at the high SiO_2_/Na_2_O ratio as compared to the low SiO_2_/Na_2_O ratio (G10M1.0 sample) in Figure 6.

**Figure 9:**
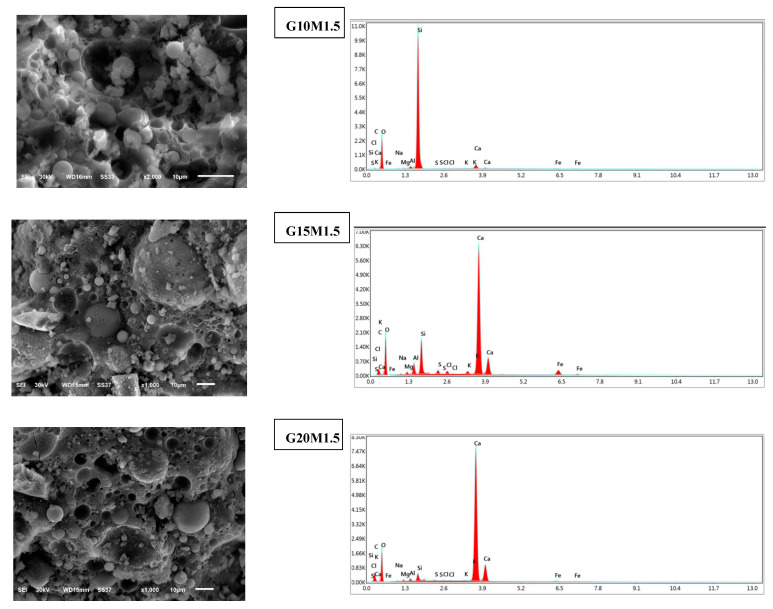
SEM images at 10 µm and chemical composition of LWGFC containing ESP at a high SiO_2_/Na_2_O ratio.

The geopolymerization products, such as N-A-S-H, C-S-H, and C-A-S-H gels, are intricately intertwined and may be detected using EDS analysis. The reason for this is that the high dissolution of GGBS and ESP greatly improved the porosity in LWGFCs by adding soluble Si to the activator [14]. This, along with raising the SiO_2_/Na_2_O ratio from 1.0 to 1.50, showed the reaction process of LWGFCs, which led to more heat release that sped up the geopolymerization process. It has been found that geopolymerization products stick to the inside walls of pores, like closed, dense pores, which helped create more barriers to lower heat transfer and strength failure. Additionally, EDS examination at a high SiO_2_/Na_2_O ratio showed the existence of several elements in the G10M1.5, G15M1.5, and G20M1.5 samples, such as O, Na, Al, Si, Ca, Mg, K, and Fe, as shown in Figure 9. O, Na, Al, Si, and Ca showed the maximum intensity elements in the high SiO_2_/Na_2_O ratio. Therefore, it is possible to infer that the bond between Na, Al, and Si atoms produced geopolymerization products [14,31]. In accordance with this, the Ca elements were dispersed similarly, although with less intensity than the Na elements. This proved that calcium silicate hydrate (C-S-H) can be made on a small scale and that it can exist with other geopolymerization products. The formation of C-S-H also aids in early strength development. Consequently, more geopolymerization products were produced when the activator ratio was higher and with proper ESP content.

### 3.4. Physic-Mechanical Properties

#### 3.4.1. Correlation Between Porosity, Oven-Dry Density, and Compressive Strength

**Figure 10:**
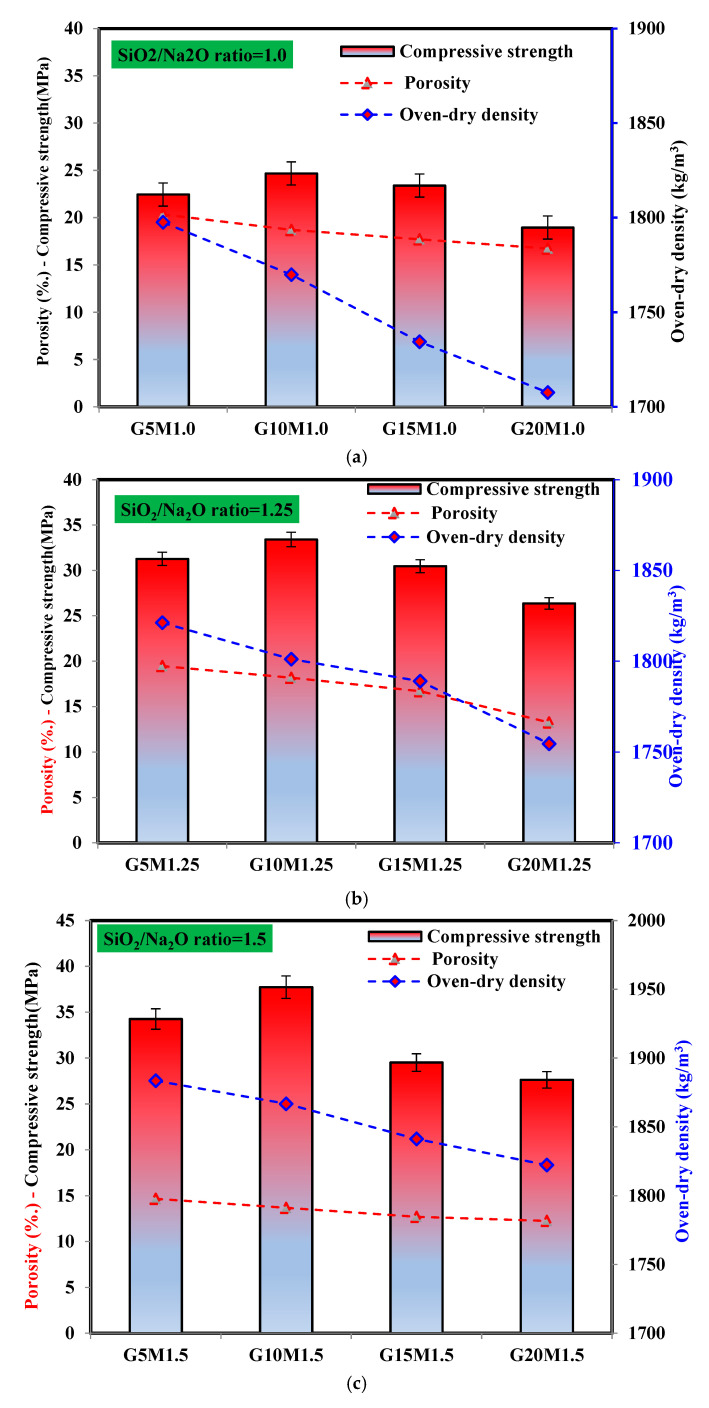
Correlation between the porosity, density, and compressive strength versus a SiO_2_/Na_2_O ratio of (**a**) 1.0, (**b**) 1.25, and (**c**) 1.5 in the LWGFC.

Figure 10 illustrates the relationship between the apparent porosity, oven-dry density, and compressive strength of LWGFC samples containing ESP at varying SiO_2_/Na_2_O ratios. At a consistent SiO_2_/Na_2_O ratio of 1.0, a reduction in density from 1797 kg/m^3^ to 1708 kg/m^3^ was observed as the ESP content increased from a 5% to 20% replacement (Figure 10a). The ESP-based LWGFC samples had low oven-dry density at a low SiO_2_/Na_2_O ratio, especially when incorporating 20%, which decreased the density up to 1707 kg/m^3^ [38,39,40]. On the other hand, the increasing of the SiO_2_/Na_2_O ratio leads to the enhancement of the kinetic reactions, which resulted in decreasing the porosity and increasing the oven-dry density. Overall, when low-density materials are added to a mixture containing Al powder, the entrapped air or meta-stable bubbles that are created mix and may even escape from the mixture’s bottom to its upper surface, expanding the volume and increasing the porosity of samples [38,47]. As a result, the increased ESP replacement exhibited a low dry density and high porosity LWGFC. As noticed in Figure 10, the addition of ESP and different SiO_2_/Na_2_O ratio changes the porosity of LWGFC samples. Medri et al. [48] and Jaya et al. [49] confirmed that the porosity degree may control the strength performance and thermal conductivity of the LWGFC. It was also noticed that the porosity and oven-dry density values were linked. This means that LWGFC with a high oven-dry density had lower porosities as well. According to Figure 10a, the porosity values of LWGFC were dropped from 20% to 16% with ESP from 5% to 20% replacement at the same SiO_2_/Na_2_O ratio, which was comparable to the 18–30% porosity value of LWGFC reported by [49]. Conversely, the current porosity was lower than the porosity (25–30%) of LWGFC reported by [50]. Similarly, with a high SiO_2_/Na_2_O ratio, the porosity also tends to decrease as the ESP content increases. In other words, the porosity of LWGFC declined from 19% to 13% and 14% to 12% with the ESP replacement from 5% to 20% at a SiO_2_/Na_2_O ratio of 1.25 and 1.5, respectively. The reason for this trend is that the ESP particle makes the geopolymerization process work better, which leads to more N-(C)-A-S-H gel filling the gaps and reducing the porosity. A similar investigation also reported that the addition of ESP to the geopolymer composite changed the pore structure and hardened properties [51]. Therefore, the changes in the oven-dry density and porosity of LWGFC were significantly associated with the content of ESP and SiO_2_/Na_2_O ratio. Both parameters were influenced by the geopolymerization process and binding skeleton, especially the SiO_2_/Na_2_O ratio, which affected the expansion volume and overall porosity.

Figure 10 also highlights the effect of varying ESP replacement levels and SiO_2_/Na_2_O ratios on the compressive strength of LWGFC after 28 days of curing. As shown in Figure 10a, a notable increase in compressive strength (approximately 14.1%) was observed when the ESP content was raised from 5% to 10% at a low SiO_2_/Na_2_O ratio of 1.0, reaching a peak strength for the G10M1.0 sample. This peak at 10% ESP can be attributed to an optimal balance between the reactivity of CaO from ESP and its pore-filling effects within the geopolymer matrix. The calcium from ESP (primarily in the form of CaCO_3_) contributes to the formation of hybrid C-(N)-A-S-H gels, enhancing early strength development by increasing the gel’s compactness, as evidenced by the reduced porosity of 18.7% for G10M1.0 (Figure 10a). Simultaneously, the fine ESP particles act as a filler, reducing micropores and improving the matrix’s density (1791 kg/m^3^ for G10M1.0), which further supports strength gain [52]. However, as the ESP content continued to increase beyond 10%, the compressive strength decreased, dropping by 8.6% (23.4 MPa) at a 15% replacement and by 26.2% (18.9 MPa) at a 20% replacement. This decline is likely due to the high calcium content, weak pozzolanic activity, high-water absorption, and a high specific surface area of ESP compared to high-activity GGBS particles, which introduce microdefects and hinder strength development at higher replacements. Paruthi et al. [19] and Rathinvael et al. [52], who examined the role of ESP in geopolymer performance, reported similar trends, noting that the chemical and mineralogical properties of ESP significantly influence strength variations. The samples with a high SiO_2_/Na_2_O ratio (Figure 10b,c) demonstrated relatively high strengths of 33.4 MPa and 37.7 MPa for G10M1.25 (density = 1801.1 kg/m^3^, porosity = 18.2%) and G10M1.5 (density = 1866.8 kg/m^3^, porosity = 13.7%), respectively. These improvements are attributed to the enhanced geopolymerization at higher SiO_2_/Na_2_O ratios, which promotes the formation of compact gels like C-A-S-H and C-S-H, further densifying the microstructure. Additionally, Tiong et al. [27] found that the inclusion of ESP in foam concrete increases compressive strength, noting that ESP’s strength contribution is comparable to that of limestone filler due to its CaCO_3_ content, which acts as an inert filler. Furthermore, Bian et al. [53] stated that a robust structure with mesopores, micropores, and a homogeneous distribution of pores within the mixture contributes to high-strength LWGFC, as will be further discussed. Therefore, the higher content of CaCO_3_ combined with a high SiO_2_/Na_2_O ratio significantly improves the strength performance of LWGFC.

#### 3.4.2. Water Absorption and Compressive Strength Relationship

**Figure 11:**
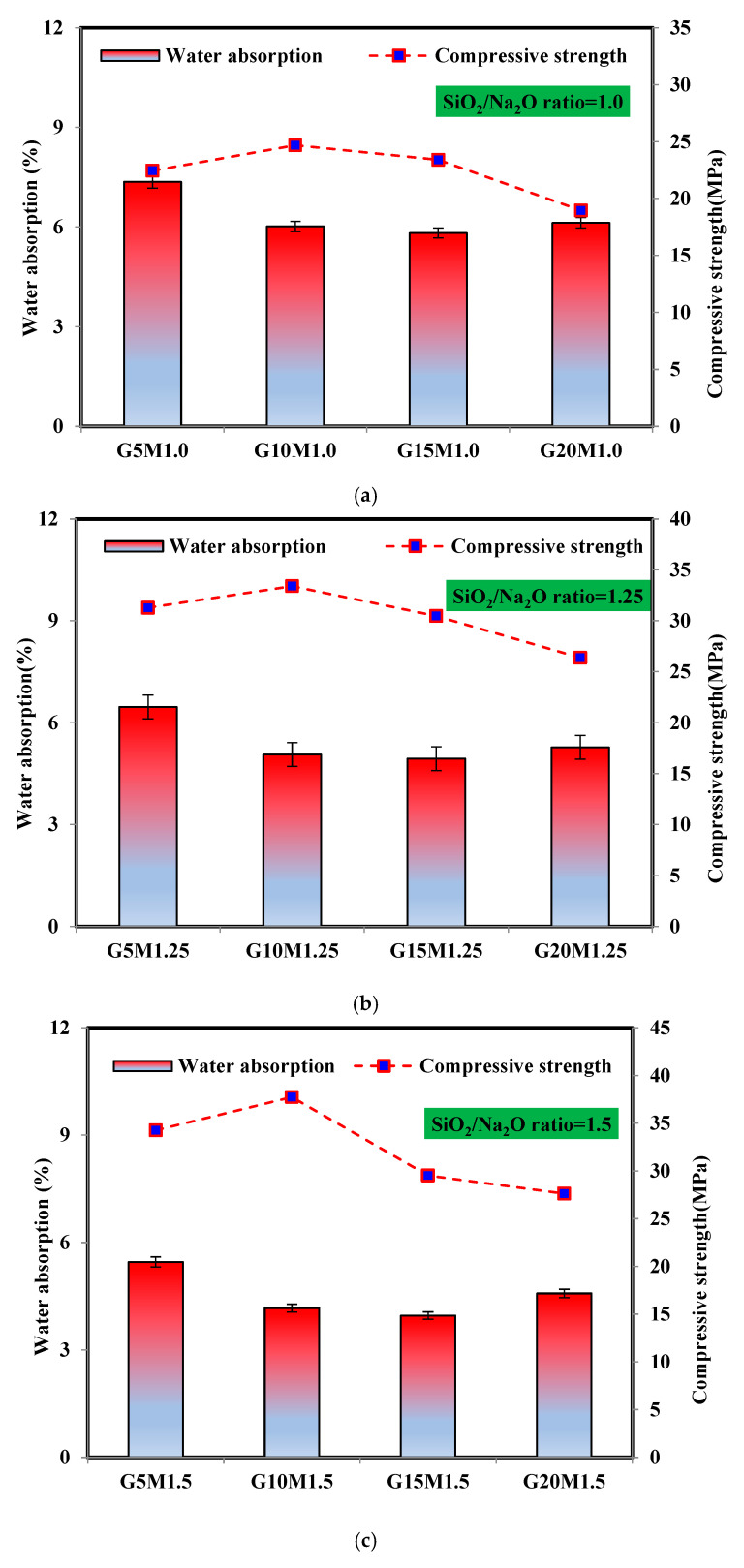
The water absorption and compressive strength of the LWGFC samples (**a**) SiO_2_/Na_2_O ratio = 1.0, (**b**) SiO_2_/Na_2_O ratio = 1.25, and (**c**) SiO_2_/Na_2_O ratio = 1.5.

Figure 11 illustrates the relationship between water absorption and compressive strength in LWGFC samples with varying SiO_2_/Na_2_O ratios and ESP content. As shown in Figure 11a, increasing the ESP content generally leads to a reduction in water absorption at a constant SiO_2_/Na_2_O ratio, except for the sample with 20% ESP content. This decrease in water absorption may be attributed to the lower porosity of these mixtures, as indicated in Figure 11a. Hence, as ESP adds extra calcium that helps to form more secondary N-(C)-A-S-H gel, it might be an excellent filler for LWGFC [20]. It is found that an increase in ESP content above 10% causes a gradual decrease in the compressive strength, as exhibited in Figure 11a. With increasing the SiO_2_/Na_2_O ratio and at the same ESP content, the water absorption was declined. The explanation for this reduction may be related to additional N-(C)-A-S-H phases that were generated by the increasing the SiO_2_ concentration. For instance, the water absorption results were decreased by 15.1% and 31.4% for G15M1.25 and G15M1.5 samples, respectively, as compared to the G15M1.0 sample. This suggests that the AAS with variable SiO_2_/Na_2_O ratios plays a key role in strength development. This rise can be demonstrated by a rise in the SiO_2_/Na_2_O ratio, which speeds up the dissolution of the GGBS and ESP particles and promotes the hydration reaction of the LWGFC. Thus, more hydration products are formed, which enhances strength development.

#### 3.4.3. Correlation Between the Thermal Conductivity Porosity and Compressive Strength

**Figure 12:**
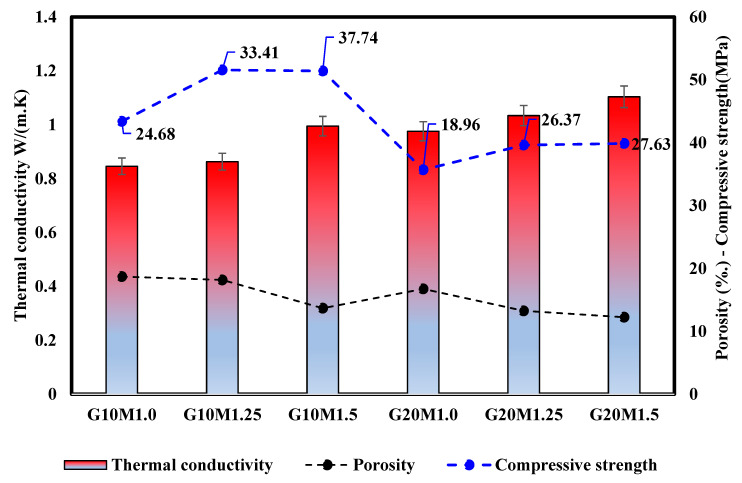
Effect of silicate modulus and ESP replacement on the thermal conductivity of LWGFC.

Figure 12 presents the results of the thermal conductivity, porosity, and compressive strength of LWGFC. Generally, it was observed that the thermal conductivity increased as the ESP replacement increased. At 10% ESP inclusion, the G10M1.0 sample exhibited lower thermal conductivity and higher porosity. As shown in Figure 12, the porosity and thermal conductivity of the G10M1.0 sample was 18.7% and 0.84 W/(m·k), respectively. In contrast, the thermal conductivity of the G20M1.0 sample increased by 27.9%, reaching 0.97 W/m·k compared to the G10M1.0 sample, both at the same SiO_2_/Na_2_O ratio. This increase in thermal conductivity may be attributed to the reduction in porosity of the G20M1.0 sample compared to the G10M1.0 sample. The higher replacement of GGBS with ESP likely promotes the formation of denser composites, as the finer surface area of ESP effectively fills the voids, leading to a more compact structure. Previous studies [33] have demonstrated that an increase in ESP content can negatively affect thermal conductivity. Additionally, for LWGFC samples with the same ESP replacement (G10M1.0 and G20M1.0), the thermal conductivity was found to increase with the rise in SiO_2_/Na_2_O ratios. For instance, the thermal conductivity G10M1.5 sample increased by 17.7% as compared with a low SiO_2_/Na_2_O ratio (G10M1.0 sample). This can be explained by the fact that the AAS is more susceptible to the SiO_2_/Na_2_O ratio and the chemical composition of the used precursor. Additionally, by adding 10% replacement and a SiO_2_/Na_2_O ratio of 1.5, LWGFC may obtain an acceptable thermal conductivity of 0.86 W/(m·k), high compressive strength (37.4 MPa), and low porosity, which encourage this type of concrete in structural applications. Finally, previous studies have demonstrated the significance of high porosity in thermal transport and thermal diffusivity [50,54]. They also confirmed that the pore characteristics and pore size distribution are responsible for the altering of thermal conductivity and density of the LWGFC, thus directly impacting the heat transfer.

**Figure 13:**
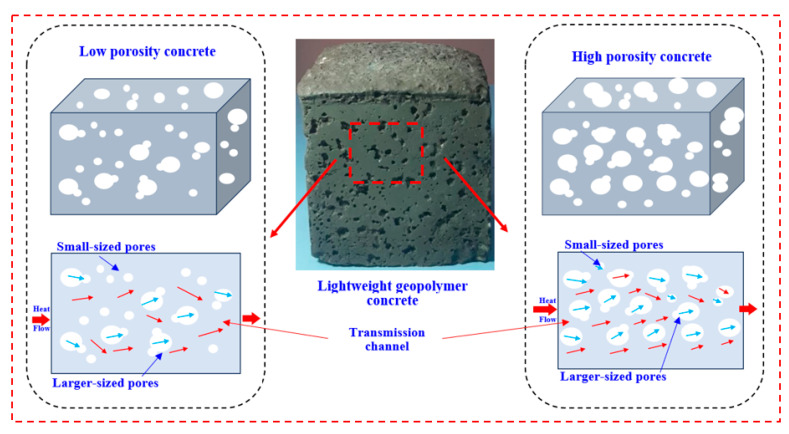
Schematic diagram of heat transfer in LWGFC.

As was previously mentioned, low densities and low thermal conductivity in LWGFC correspond to high porosities, which may be lower than compressive strength. Therefore, Figure 13 shows the schematic diagram of heat transport in both high and low porosity systems. At a low SiO_2_/Na_2_O ratio, the high porosity in LWGFCs can form the route for thermal transfer, which is associated with the high liquid content in these mixtures and resulted in decreasing thermal conductivity. Contrarily, the increasing of the SiO_2_/Na_2_O ratio reduced the large pores as recommend by SEM micrographs, which is the reason that thermal conductivity increased. As a result, the decreasing of the SiO_2_/Na_2_O ratio tends to mitigate the thermal transfer effectively. Thus, the high number of pores associated with low SiO_2_/Na_2_O ratios and low content of ESP replacement leads to resistance of thermal transfer. Additionally, Figure 13 makes it abundantly evident that the high porosity and thermal conductivity are positively correlated, while the effect of the porosity has a significant effect on the hardening properties and the microstructure of the LWGFC.

#### 3.4.4. Comparison with LWGFC Properties in Previous Studies

**Figure 14:**
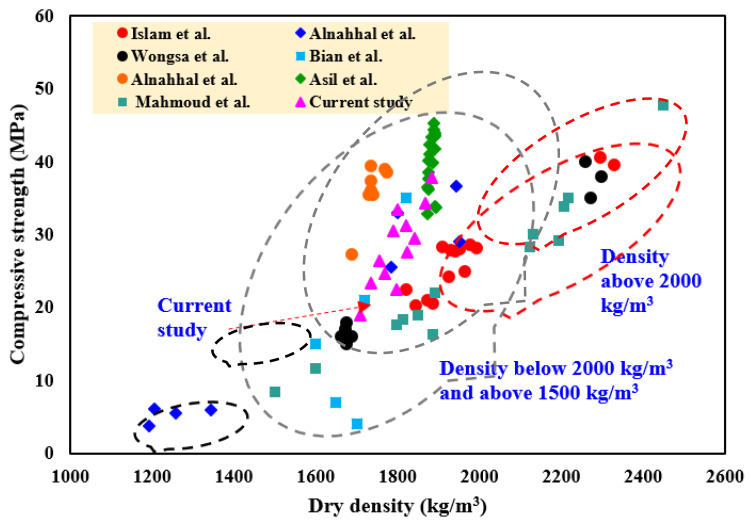
Comparison of the density and compressive strength of LWGFC with previous studies [50,53,56,57,58,59,60].

**Figure 15:**
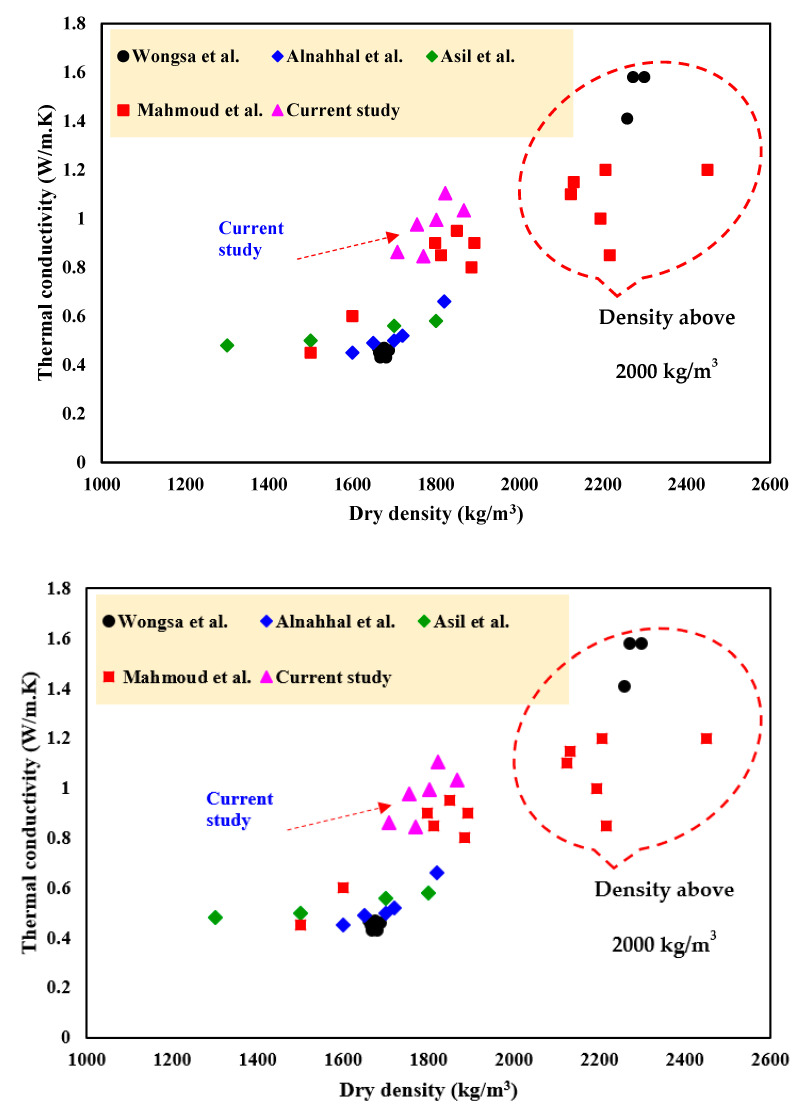
Comparison of the density and thermal conductivity of LWGFC with previous studies [57,58,59,60].

Figure 14 and Figure 15 summarize previous works to aid in understanding the pattern of variation in thermal conductivity, dry density, and strength development of LWGFC with different densities [20,51,53,55,56,57,58,59,60]. In previous studies, it was demonstrated that lightweight concrete promotes low thermal properties and strength development, making it a viable option for thermal insulation applications. Thus, one of the most important indicators for regulating the mechanical and thermal properties of LWGFC is its dry density. The results in Figure 14 and Figure 15, extracted from earlier research, demonstrate a similar tendency to the current investigation, where LWGFC with an oven-dry density greater than 1600 kg/m^3^ is likely suitable for structural applications. The current study achieved thermal conductivity values ranging from 0.84 W/m·K (G10M1.0) to 0.97 W/m·K (G20M1.0), as shown in Figure 15, which are competitive when contrasted with conventional lightweight concretes and insulation materials. For instance, traditional lightweight concretes, such as those made with expanded clay or perlite, typically exhibit thermal conductivities of 0.9–1.2 W/m·K at similar densities (1600–1800 kg/m^3^) [56,60], while common insulation materials like expanded polystyrene (EPS) and mineral wool have lower values of 0.03–0.04 W/m·K and 0.035–0.045 W/m·K, respectively, but lack structural capacity. The LWGFC in this study, with a thermal conductivity of 0.84–0.97 W/m·K, offers a balanced compromise, providing better insulation than conventional lightweight concretes while maintaining sufficient compressive strength (e.g., 37.4 MPa at 10% ESP and SiO_2_/Na_2_O = 1.5) for structural use, making it a practical choice for energy-efficient building applications where both insulation and load-bearing capacity are required. Referring to Figure 15, the thermal conductivity and density of LWGFC were associated with its porosity, bonding skeleton characteristics, and the type and content of the used precursors. Additionally, the proper choice of precursor content and alkali-activated solution (AAS) resulted in reduced porosity and positively affected the strength properties of LWGFC. Conversely, the high porosity of LWGFC contributes to lowering the density while deteriorating the compressive strength, as presented in Figure 15. The current investigation confirmed that thermal conductivity decreases with a decrease in dry density, which is likely related to the pore structure in the matrix. Additionally, the increased compressive strength of LWGFC is associated with higher densities, owing to the excellent skeleton bonding in the matrix. Finally, the density of the LWGFC in this study is lower than that of LWGFC produced by [55,59], with comparable strength.

## 4. Conclusions

In the current investigation, the effect of different ESP replacements and SiO_2_/Na_2_O ratios on the expansion behavior, strength performance, and microstructure of the LWGFC were examined. Overall, the optimum ESP content and SiO_2_/Na_2_O ratios reduced the porosity with acceptable thermal conductivity results and improved the compressive strength. Accordingly, the results obtained can be summarized as follows:

1. The inclusion of ESP as a partial replacement for GGBS accelerated the setting time due to its high CaCO_3_ content and high-water absorption. Further, by increasing the SiO_2_/Na_2_O ratio to 1.25 and 1.5, the setting time declined by 12.07% and 27.59%, respectively, compared to the SiO_2_/Na_2_O ratio of 1.0.
2. At all SiO_2_/Na_2_O ratios, the optimum content of ESP in LWGFC was 10%, which reduced the porosity by 2.89% and 26.94% and enhanced the compressive strength by 35.37% and 52.92% at SiO_2_/Na_2_O ratios of 1.25 and 1.5, respectively, compared to the low SiO_2_/Na_2_O ratio, which was highly associated to the geopolymerization reaction of LWGFC samples containing optimum ESP.
3. The thermal conductivity was negatively affected by the ESP incorporation SiO_2_/Na_2_O ratio, which related to the decreasing porosity and increased oven-dry density. For instance, the thermal conductivity of samples with 20% ESP increased by 15.3% compared to 10% ESP samples at a low SiO_2_/Na_2_O ratio. Further, increasing the SiO_2_/Na_2_O ratio could improve the microstructure of LWGFC, thus leading to increased thermal conductivity. This enhancement may be due to the high dissolution of rich-calcium materials (GGBS and ESP) in the high alkaline-activation solution, which generates more geopolymerization reactions and consequently tends to reduce LWGFC porosity.

The findings of this study on LWGFC with varying ESP content and SiO_2_/Na_2_O ratios provide valuable insights into its potential for sustainable construction applications, but several areas warrant further investigation to optimize performance and broaden its practical use.

- Firstly, while ESP incorporation up to 10% enhances the formation of N-(C)-A-S-H gels, improving compressive strength and reducing porosity, higher ESP contents (e.g., 20%) lead to a significant strength reduction (up to 26.2%) due to its high calcium content, weak pozzolanic activity, and high-water absorption. Future studies should explore hybrid additives, such as combining ESP with other pozzolanic materials like silica fume or nano-silica, to mitigate the negative effects of high ESP replacement while maintaining its sustainability benefits. This approach could enhance the pozzolanic reactivity and reduce microcracking, potentially achieving a better balance between strength and density.
- Secondly, the increase in thermal conductivity with higher ESP content (e.g., from 0.84 W/m·K at 10% ESP to 0.97 W/m·K at 20% ESP) and SiO_2_/Na_2_O ratios (e.g., a 17.7% increase from SiO_2_/Na_2_O = 1.0 to 1.5) indicates a trade-off between mechanical strength and thermal insulation properties. To address this, future research should investigate the incorporation of additional lightweight fillers or foaming agents that can maintain low thermal conductivity while preserving the strength gains achieved at higher SiO_2_/Na_2_O ratios. For instance, materials like perlite or expanded polystyrene beads could be tested to further reduce density and thermal conductivity, making LWGFC more suitable for energy-efficient building applications.

## Figures and Tables

**Table 1:** Chemical oxides in binders.

Oxides (%)	GGBS	FA	SF	ESP
CaO	36.83	7.6	0.35	94.04
SiO_2_	39.6	46.44	96.29	0.342
Al_2_O_3_	13.79	38.10	0.2	-
TiO_2_	0.58	1.17	-	-
Fe_2_O_3_	1.69	3.12	0.53	-
P_2_O_5_	-	0.76	-	2.09
MgO	5.79	0.23	0.66	1.22
SrO	-	-	-	0.48
SO_3_	-	0.69	0.17	1.15
K_2_O	0.97	0.88	0.56	0.2
Na_2_O	0.48	0.4	0.38	0.4
L.O.I	0.32	-	-	2.4
Basicity Index	0.98	-	-	-

**Table 2:** Mixture proportion of LWGFC.

Mix Type	kg/m^3^									(%)	SiO_2_/Na_2_O
	GGBS	SF	FA	ESP	Sand	Dolomite	NaOH	Na_2_SiO_3_	Extra Water	Al Powder	
G5M1.0	295	57	76	4.8	459	846	83	195	108	0.9	1.0
G10M1.0	278			9.6			82	195			
G15M1.0	264			14.4			82	195			
G20M1.0	249			19.2			82	195			
G5M1.25	295			4.8			79	242	86		1.25
G10M1.25	278			9.6			77	242	85		
G15M1.25	264			14.4			77	242	83		
G20M1.25	249			19.2			77	242	81		
G5M1.5	295			4.8			62	273	72		1.5
G10M1.5	278			9.6			60	273			
G15M1.5	264			14.4			59	273	71		
G20M1.5	249			19.2			58	273			

## Data Availability

The original contributions presented in this study are included in the article. Further inquiries can be directed to the corresponding authors.
